# Spider angioma at the injection site of the meningitis vaccine

**DOI:** 10.1093/omcr/omae001

**Published:** 2024-02-16

**Authors:** Hadi Alabdullah, Maher Almousa, Muhammad Nour Alabdullah, Ahmad Zahi Al-shawaf, Thaer Douri

**Affiliations:** Faculty of medicine, University of Hama, Hama, Syrian Arab Republic; Faculty of medicine, University of Hama, Hama, Syrian Arab Republic; Otorhinolaryngology Department, Al-Mowassat University Hospital, Faculty of Medicine, Damascus University, Damascus, Syrian Arab Republic; Department Of Pathology, Faculty of dentistry, Al-Wataniya Private University, Hama, Syrian Arab Republic; Department of Dermatology, Alassad Medical Centre, Hama, Syrian Arab Republic

## Abstract

Spider angioma is a cutaneous nevus caused by a vascular abnormality, characterized by a central red area with radiating reddish, web-like extensions. It is typically associated with liver cirrhosis, hyperestrogenism, and alcohol consumption. In this case report, we present a unique instance of a patient who developed spider angioma at the injection site of the meningitis vaccine as a long-term adverse effect. The lesion was treated with electrocautery and diminished within one week of treatment. This case highlights the potential for spider angioma to develop as a long-term adverse effect of the meningitis vaccine, a possibility that has not been previously reported. Further research is required to understand the underlying mechanisms and identify potential risk factors for this rare adverse effect.

## INTRODUCTION

Spider angioma, also known as spider nevus, is a benign vascular anomaly that may appear as a single or multiple lesions. This lesion is characterized by a central red spot with reddish, web-like extensions spreading outwards. It is typically asymptomatic and resembles a spider’s body. These lesions are commonly found in areas supplied by the superior vena cava and usually measure less than 2 cm [1]. The most common side effects of the meningitis vaccine include pain, redness, or swelling at the injection site; however, these symptoms are generally mild and resolve within a few days [2]. In this report, we present a case of spider angioma that developed at the meningitis vaccine injection site as a rare long-term adverse effect.

## CASE REPORT

**Figure 1:**
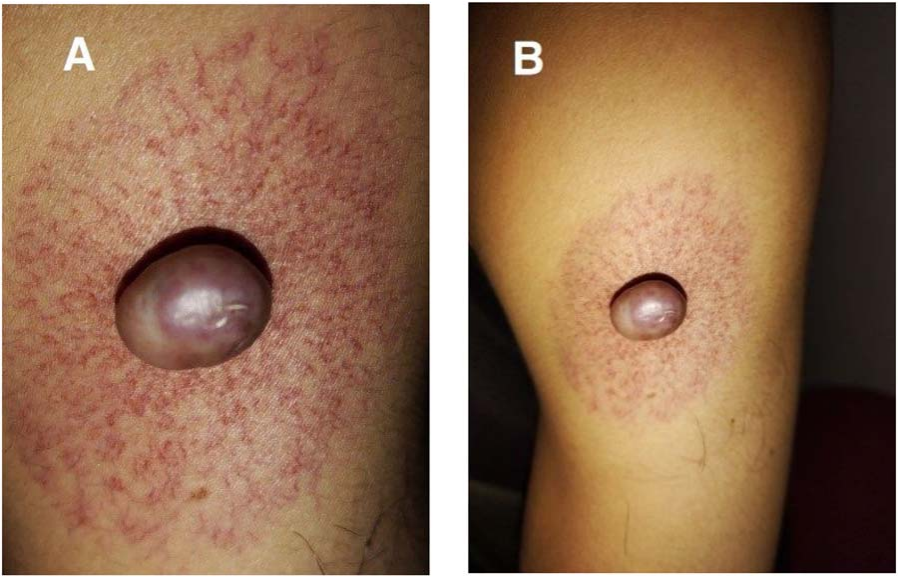
(**A** and **B**) Photograph images of the lesion showing parts of the nevus (body and legs).

**Figure 2:**
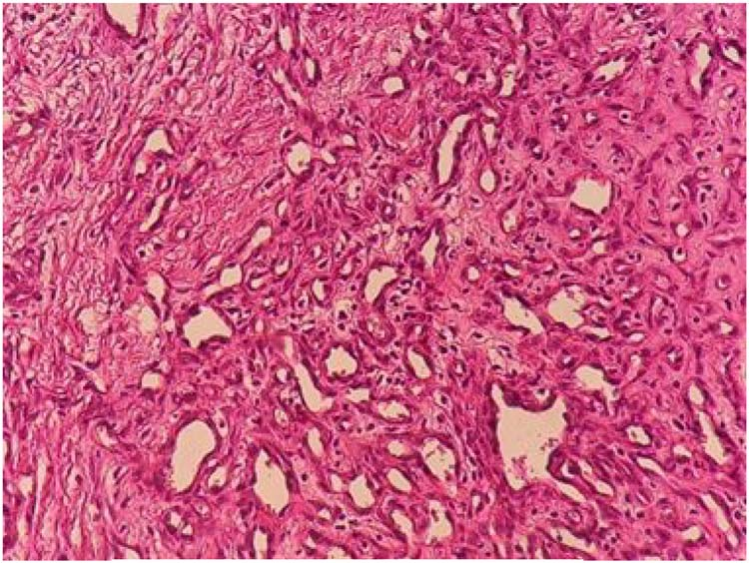
Image of histopathology of lesion with H&E staining shown dilated vessels of various sizes have proliferated in the superficial and mid-dermis. Inflammatory cells, mainly lymphocytes and histiocytes, infiltrated around the dilated vessels, and erythrocyte extravasation.

**Figure 3:**
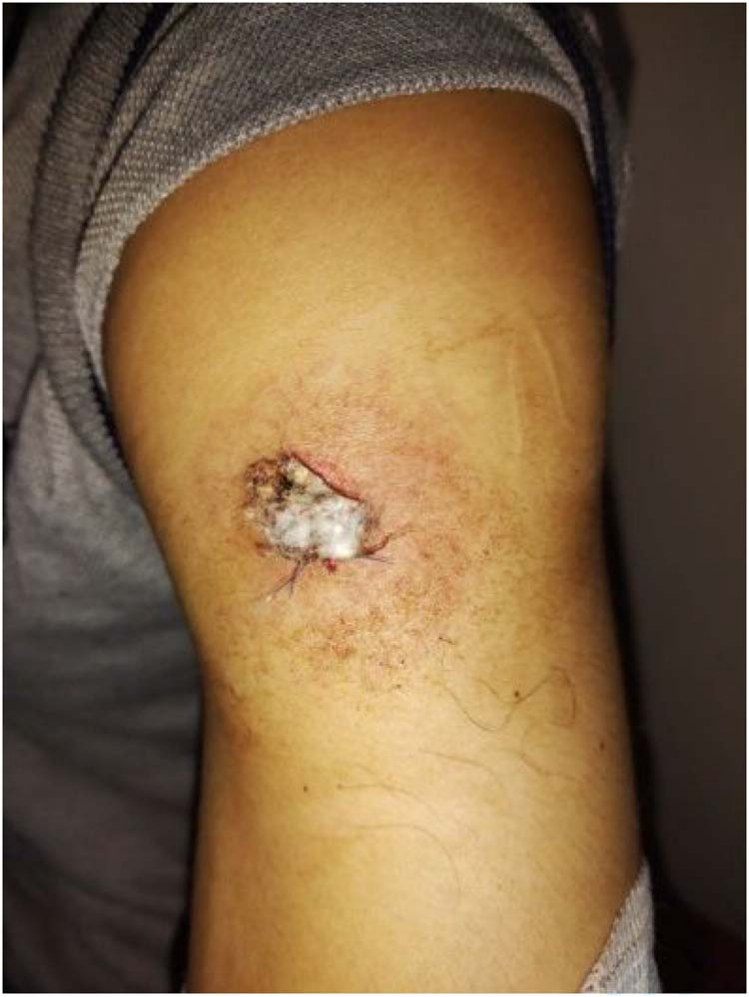
Photograph image of the lesion after a week of treatment showing diminished lesion.

**Figure 4:**
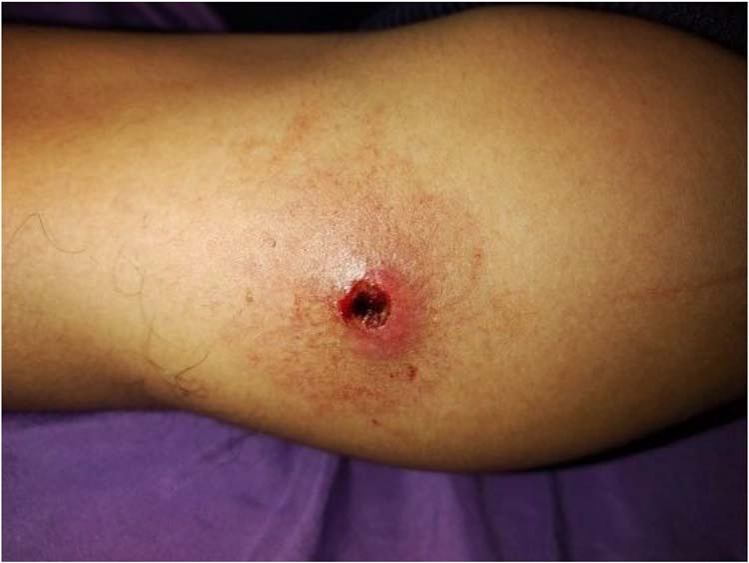
Photograph image of the lesion two weeks after treatment showing diminished lesion.

A 28-year-old male was admitted to the Department of Dermatology with complaints of a large reddish-purple, soft tumor featuring radiating telangiectasia on the left upper arm, Specifically above the deltoid muscle (Fig. 1A and B). He exhibited no additional symptoms or relevant medical history. The lesion had been developed for over ten years, appearing subsequent to preventative vaccination for meningitis. Upon physical examination, the lesion resembled a keloid but was soft upon palpation. Laboratory tests were negative for hepatitis B and C. Liver enzymes and other laboratory parameters were within normal ranges. A skin biopsy was obtained from the central reddish-purple area of the lesion. Histopathological analysis revealed variously sized dilated vessels proliferating in the superficial and mid-dermis. There was infiltration of inflammatory cells, predominantly lymphocytes and histiocytes, around the dilated vessels, with evidence of erythrocyte extravasation noted in the area (Fig. 2). The lesion was treated with electrocautery, and within one week of treatment, the lesion had diminished (Fig. 3). After two weeks, the lesion had diminished even further than before (Fig. 4).

## DISCUSSION

Spider angioma is a type of cutaneous nevus caused by a vascular abnormality. It is characterized by a central red spot with radiating reddish, spiderweb-like extensions. A typical spider angioma is made up of a central body, radial ‘legs,’ and an erythematous halo surrounding it. The central body is a painful arteriole, between 1 to 10 mm in diameter. The ‘legs’ consist of a larger arteriole and numerous smaller blood vessels that extend outward from the central body like a spider’s legs, creating a pattern of telangiectasia. Spider angiomas usually measure less than 2 cm, though there have been rare instances of very large spider angiomas, such as the case being reported. Spider angiomas typically appear in areas supplied by the superior vena cava, including the neck, arms, face, and upper chest [1, 2].In this instance, the nevus was located on the upper arm. The precise cause of spider angiomas remains uncertain, but they have been linked to conditions such as liver cirrhosis, hyperestrogenism, and excessive alcohol consumption. They have also been observed in patients with thyrotoxicosis, rheumatoid arthritis, and those using oral contraceptives [4]. A suggested mechanism underlying the formation of spider nevi is the angiogenesis driven by elevated blood levels of vascular growth factors, notably vascular endothelial growth factor (VEGF) and basic fibroblast growth factor (bFGF), in patients with liver cirrhosis [5].Solitary, spider angiomas are observed in 15% of young adults and typically present with fewer than three lesions [6]. Our patient developed a spider angioma that appeared to be an isolated case; however, it emerged at the same site as a meningitis vaccine injection administered 10 years earlier when the patient was 18 years old. Side effects of meningitis vaccines tend to be mild and resolve within a few days. The most common side effects include pain, redness, or swelling at the injection site [3].

The management of spider angioma is contingent upon its underlying conditions, as it is indicative of systemic diseases. Consequently, treating these underlying conditions ought to be the primary objective. In isolated instances, spider angioma can be addressed using laser electrocautery, electro-desiccation or Surgery [6]. Our patient underwent treatment with electrocautery, and the follow-up after one week fell within normal limits. Two weeks post electrocautery treatment, the results showed marked improvement.

## CONCLUSION

Spider angioma may indicate underlying conditions such as liver cirrhosis or pregnancy. Solitary cases of spider angioma in healthy individuals have also been observed. The development of spider angioma following meningitis vaccination has never been reported before, making this case unique in suggesting a possible long-term side effect of the meningitis vaccine.

## CONFLICT OF INTEREST STATEMENT

The authors declare that they have no conflicts of interest.

## FUNDING

This research did not receive any external or internal funding.

## ETHICAL APPROVAL

Not applicable.

## PATIENT CONSENT

Written informed consent was obtained from the patient to publish this case report and any accompanying images.

## GUARANTOR

Maher Almousa and Hadi Alabdullah.
